# Comparison of visual outcomes after implantation of AtLisa tri 839 MP and Symfony intraocular lenses

**DOI:** 10.1007/s10792-020-01435-z

**Published:** 2020-06-02

**Authors:** Wojciech Lubiński, Karolina Podborączyńska-Jodko, Marta Kirkiewicz, Maciej Mularczyk, Michał Post

**Affiliations:** 1 2nd Chair and Department of Ophthalmology, Pomeranian Medical University, Powstańców Wlkp. 72, 70-111 Szczecin, Poland; 2 Chair and Department of Basic and Clinical Anatomy, Pomeranian Medical University, Szczecin, Poland

**Keywords:** Multifocal lens, Trifocal lens, Extended depth of focus, Diffractive, Aspheric, Visual function, EDOF

## Abstract

**Purpose:**

To compare visual outcomes after implantation of AtLisa tri 839 MP and Symfony intraocular lenses (IOLs).

**Methods:**

All subjects underwent sequential bilateral cataract extraction with AtLisa tri 839 MP or Symfony IOL implantation. The design is prospective case series. Each group consists of 20 patients (40 eyes). At 1 year postoperatively, the following parameters were analysed: binocular uncorrected visual acuity (log MAR): for distance (UDVA) at 4 m, for intermediate distances (UIVA) at 60, 70, 80 cm and for near (UNVA) at 40 cm, defocus curve, mesopic and photopic contrast sensitivities (CSs), spectacle independence, visual function test questionnaire modified VFQ-25), photopic phenomena and postoperative complications.

**Results:**

In the AtLisa tri 839 MP group, the mean binocular UNVA and UIVA were significantly better than in the Symfony group (UNVA: − 0.01 ± 0.04 vs. 0.21 ± 0.15; *p* = 0.000; 60 cm UIVA: − 0.01 ± 0.04 vs. 0.09 ± 0.09, *p* = 0.001; 70 cm UIVA − 0.05 ± 0.06 vs. 0.11 ± 0.08, *p* = 0.002; 80 cm UIVA − 0.01 ± 0.06 vs. 0.15 ± 0.08, *p* = 0.019). There were no significant between-group differences in the mean binocular UDVA and CS, with one exception: the mean binocular distance CS (18 cpd) under mesopic conditions was significantly better in the Symfony group than in the AtLisa tri 839 MP group (1.39 ± 0.22 vs. 1.17 ± 0.27; *p* = 0.015). The defocus curve analysis revealed significant between-group differences at vergences of 2.0 to − 4.0 D (*p* < 0.05), except for 2.0, 1.0, 0 and − 1.5. All subjects in AtLisa tri 839 MP group and 18 subjects (90%) in Symfony group were spectacle independent. Patients from both groups highly rated their overall vision quality in the VFQ-25 (1.67 ± 0.47 vs. 1.85 ± 0.5 in the Symfony and AtLisa tri 839 MP group, respectively, *p* = NS). The scores for daytime driving (1.00 ± 0.00 vs. 1.21 ± 0.36; *p* = 0.002), night driving (1.57 ± 0.55 vs. 2.13 ± 1.15; *p* = 0.027) and difficult situation driving (1.14 ± 0.31 vs. 1.53 ± 0.56; *p* = 0.049) were significantly better in the AtLisa tri 839 MP group than in the Symfony group. The incidence and perception level of halo and glare were significantly reduced (*p* = 0.00) in the Symfony group as compared to the AtLisa tri 839 MP group. The postoperative course was uneventful in all subjects.

**Conclusions:**

Visual outcomes achieved with both IOLs are comparable. In both groups, 90% of patients achieved spectacle independence. Whereas the AtLisa tri 839 MP IOL implantation was associated with slightly better intermediate distance VA and significantly better near VA, photic phenomena were less perceived by patients with Symfony IOLs.

## Introduction

A selected group of patients with cataract or presbyopia can benefit from multifocal intraocular lens (IOLs) implantation. Trifocal IOLs work by splitting the light into three separate points of focus, thus correcting distance, intermediate distance and near vision. This IOL implantation enables achieving satisfying visual acuity for all distances [1–5]. However, its disadvantages include loss of contrast sensitivity and unwanted photopic phenomena, glare and halo.

Currently, there are several trifocal diffractive IOLs available on the market, such as the FineVision (PhysIOL SA) [6], the AcrySof IQ Panoptix (Alcon), the Acriva Reviol Tri-ED 611 (VSY Biotechnology), the Versario 3F (Bausch & Lomb) and the AtLisa tri (Carl Zeiss AG) [7].

Another possibility to restore good vision for all distances is to implant IOLs with an extended depth of field (EDOF), such as Symfony (Abbott Park, AT LARA 829 MP-Carl Zeiss AG). The diffractive optics of Symfony IOL provides a single elongated focal zone as opposed to two distinct points of focus. The new technology relies on a biconvex wavefront-designed anterior aspheric surface and a posterior achromatic diffractive surface to correct chromatic aberration for enhanced contrast sensitivity with an echelette design feature to extend the range of vision. The potential superiority of EDOF lenses in comparison with trifocal IOLs involves fewer photopic phenomena and better intermediate vision. Thus, these lenses are particularly attractive to patients with an active lifestyle, who wish to be spectacle independent for most of the days but are more sensitive to halo and glare.

The literature review yields only a few studies which compared visual outcomes after bilateral implantation of EDOF IOLs and trifocal IOLs (Symfony vs. Panoptix, Symfony vs. FineVision, PanOptix vs. others) [8–14]. Both IOLs provided satisfying distance and intermediate distance vision, but trifocal IOLs were better for patients with near vision requirements. A relatively higher frequency or greater degree of bother of photic phenomena (glare, halo) is reported with Symfony IOL than with trifocal IOLs and PanOptix IOL [8, 14]. To date, there is only one 3-month follow-up comparison of visual outcomes after implantation of Symfony and AtLisa tri 839 MP [11].

That is why we decided to analyse visual outcomes and patient satisfaction after bilateral implantation of those two lenses.

## Methods

The prospective study included patients after uneventful, sequential, bilateral cataract surgery at the 2nd Department of Ophthalmology in Szczecin, Poland. All patients wanted to become spectacle independent and were informed about advantages and disadvantages of multifocal IOL implantation. The study adhered to the Declaration of Helsinki and was approved by the local ethics committee. Written informed consent was obtained from all patients.

The inclusion criteria for multifocal IOL implantation included: age of 38–70 years, bilateral cataract, pupil size 3–6 mm in dim light, preoperative corneal astigmatism below 0.75 D, hyperopic presbyopia, patient motivation for spectacle independence, tolerance of imprecise vision, willingness and ability to comply with scheduled visits.

The exclusion criteria included: ocular diseases other than cataract, high myopia with axial length (AL) ≥ 25,5 mm and hyperopia (AL ≤ 21.5 mm), lifestyle and work-related factors, such as unrealistic visual expectations, patients demanding visual precision, i.e. pilots, professional drivers, architects, etc., patients satisfied with reading glasses, individuals over 70 years of age due to likely difficulties with neuroadaptation problems to new optical conditions, as well as personality-related factors, such as mental disorder of any type, patients unsatisfied with progressive spectacle lens, history of stroke and dyslexia.

### Intraocular lenses

AtLisa tri 839 MP IOL (Carl Zeiss Meditec AG, Jena, Germany) is an acrylate, diffractive, trifocal, preloaded hydrophilic (water content of 25%) IOL with hydrophobic surface (7) with a 6.0-mm biconvex optic, an overall length of 11 mm and a posterior surface with asphericity of − 0.18. It has a 4-haptic design with a 0 degree angulation and the refractive index of 1.46. The IOL optics has a 360-degree square edge to prevent posterior capsule opacification. The near power of this lens is + 3.33 D and an intermediate power + 1.66 D in the lens inner 4.34 mm trifocal area and + 3.75 D add in its outer bifocal area. It is available in the range of spherical powers between 0 and 32 D in 0.5 D increments. The light distribution is 50% to distance, 20% to intermediate distance and 30% for near. The manufacturer’s A-constant for this IOL is 118.6.

The Tecnis Symfony IOL (Abbot Medical Optics, Santa Ana, CA) is a C–loop IOL with 6 mm optical diameter and overall diameter of 13 mm with a refractive index of 1.47. The anterior surface of the its optics is aspheric (− 0.27 µm) and the posterior surface is diffractive, which compensates for the eye’s chromatic aberrations and increases the depth of focus. The diffractive design known as echelette is pupil independent. It only enhances contrast sensitivity using achromatic technology. The Tecnis Symfony is an Extended Depth of Focus lens (EDOF). It works by creating a single elongated focal point to enhance the range of vision or the depth of focus. This lens is available in the range of spherical powers between 5.0 and 34 D in 0.5 D increments.

### Preoperative and postoperative examination

Preoperatively, all patients underwent a comprehensive ophthalmic examination, including uncorrected and best corrected visual acuity, subjective refraction, corneal topography (Atlas 9000, Carl Zeiss Meditec AG), slit lamp biomicroscopy of the anterior and posterior segments Volk lens, Pascal tonometry and biometry (IOL Master 500, Carl Zeiss Meditec AG).

Similarly, the clinical evaluation performed in all subjects 12 months postoperatively included the following: uncorrected distance visual acuity (UDVA) (logMAR ETDRS chart at 4 m), uncorrected near visual acuity (UNVA) (logMAR at 40 cm), uncorrected intermediate visual acuities (UIVA) at 60, 70, 80 cm, defocus curve with ETDRS chart positioned at 6 m assessed under photopic conditions [the change of VA addition of 0.5 D increments at each step towards hyperopia (0 to + 2.0 D) and myopia (0 to − 4.0 D)], distance (2.5 m) contrast sensitivity assessed under photopic (85 cd/m^2^) and mesopic (3 cd/m^2^) conditions, near (35 cm) contrast sensitivity assessed under photopic conditions (1.5, 3, 6, 12, and 18 cycles/degree, CSV-1000, Functional Acuity Contrast Test—FACT), spectacle independence, photic phenomena (glare and halo), patient satisfaction (modified Visual Function Questionnaire VFQ-25) [15] and postoperative complications.

### Surgical technique

Phacoemulsification (Stellaris, Bausch & Lomb) was performed in both groups by the same surgeon (WL) under topical (alcaine) and intraocular (1% lidocaine) anaesthesia. Symfony IOL was implanted through a mean 2.2-mm clear corneal incision and the AtLisa tri 839 MP IOL through a mean 1.8-mm clear corneal incision. The size of capsulorhexis was approximately 5 mm. Cataract extraction was followed by intracapsular IOLs implantation. The IOL power was calculated using optical biometry (IOL Master, Carl Zeiss Meditec, Jena, Germany) and the SRK-T (for axial length from 22 to 24.5 mm) or Hoffer Q (axial length below 22 mm) formulas. The target refraction was emmetropia.

Postoperatively, all patients were prescribed moxifloxacin, prednisolone and nepafenac eye drops for the first 4 weeks.

### Statistical analysis

The normality of distribution was verified using the Shapiro–Wilk test. As the obtained data were not normally distributed, the between-group comparisons were made using the Mann–Whitney *U* test. The results were considered statistically significant at *p* < 0.05. All analyses were performed using Statistica 12 software bundle.

## Results

AtLisa tri 839 MP IOLs were implanted binocularly in 40 eyes of 20 patients (12 women, 8 men). Symfony IOLs were implanted binocularly in 40 eyes of 20 patients (12 women, 8 men). The mean age in the AtLisa tri 839 MP group was 55.0 ± 7.1 years (range 42–69 years) and did not differ significantly (*p* = 0.422) from the mean age in the Symfony group (62.4 ± 7.9; range 38 − 68 years).

Preoperatively, the mean BCVA in the AtLisa tri 839 MP group was 0.30 ± 0.29 logMAR, and the mean axial length was 23.68 ± 0.67. Preoperatively, the mean BCVA in the Symfony group was 0.31 ± 0.21 logMAR, and the mean axial length was 23.94 ± 0.43 (*p* > 0.05).

Postoperatively, the mean residual sphere, residual cylinder and spherical equivalents in the AtLisa tri 839 MP group were − 0.10 ± 0.17 D, − 0.28 ± 0.14 D and − 0.30 ± 0.22 D, respectively, as compared to − 0.22 ± 0.35 D, − 0.39 ± 0.38 D and − 0.45 ± 0.36 D, respectively, in the Symfony group.

### Visual outcomes

There was no significant difference between the mean binocular UDVA (− 0.12 ± 0.1 logMAR vs. 0.08 ± 0.08 logMAR in AtLisa tri 839 MP vs. Symfony group, *p* = 0.261).

**Fig. 1:**
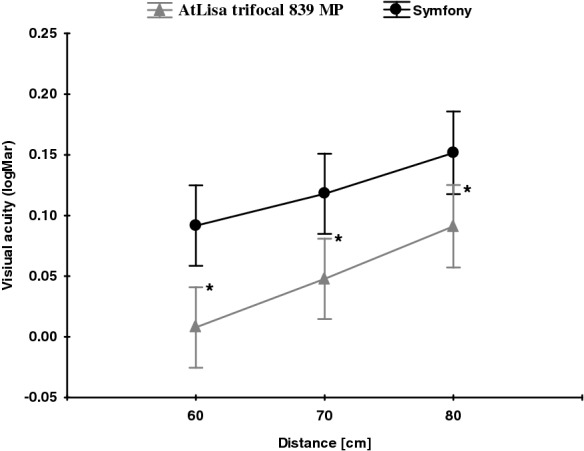
Mean binocular UIVA at 60, 70, 80 cm in both groups; significant differences were marked with an asterisk

In the AtLisa tri 839 MP group, the mean binocular UNVA and UIVA were significantly better than in the Symfony group [UNVA: − 0.01 ± 0.04 logMAR vs. 0.21 ± 0.15 logMAR; *p* = 0.000; 60 cm UIVA: − 0.01 ± 0.04 LogMAR vs. 0.09 ± 0.09 logMAR, *p* = 0.001; 70 cm UIVA − 0.05 ± 0.06 logMAR vs. 0.11 ± 0.08 logMAR, *p* = 0.002; 80 cm UIVA − 0.01 ± 0.06 logMAR vs. 0.15 ± 0.08 logMAR, *p* = 0.019] (Fig. 1).

### Defocus curves

**Fig. 2:**
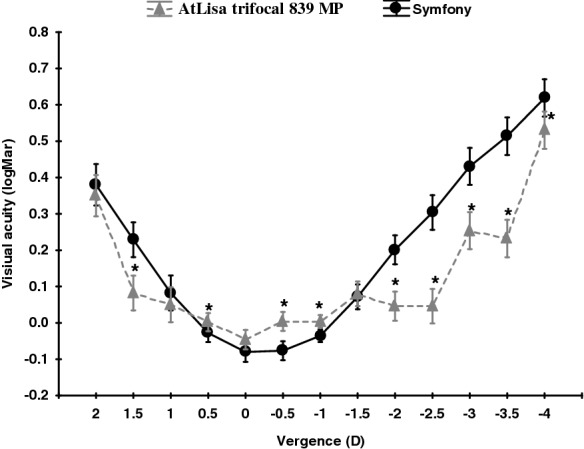
Binocular defocus curves in AtLisa tri 839 MP and Symfony groups. All data were presented as a mean ± SD. *p < 0.05

Binocular defocus curves in both groups are shown in Fig. 2.

Significant differences between AtLisa tri 839 MP and Symfony group defocus curves were detected for the following vergences: 1.5; 0.5; − 0.5; − 1.0; − 2.0; − 2.5; − 3.0; − 3.5 and − 4.0 D (*p* < 0.05).

### Spectacle independence

All subjects in AtLisa tri 839 MP group and 18 subjects (90%) in Symfony group were spectacle independent. Two subjects from Symfony group reported near vision difficulty and needed additional prescription (add + 1.0 D) to see small letters.

### Contrast sensitivity

**Fig. 3:**
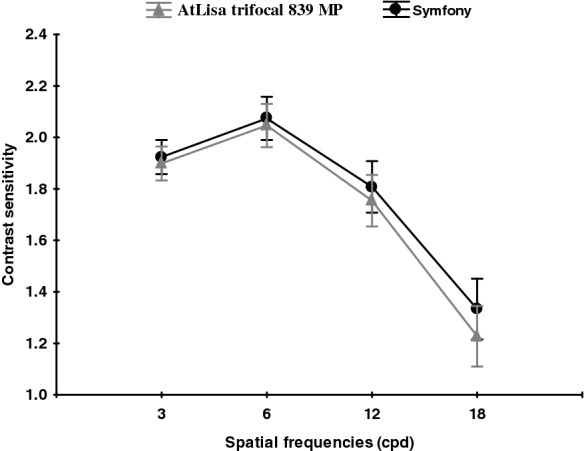
Mean binocular distance contrast sensitivity under photopic conditions in both groups. A comparison between the Symfony (round black points) and Trifocal (triangular grey points) lenses under photopic conditions. All data are presented as a mean (points) and 95% confidence interval (whiskers)

**Fig. 4:**
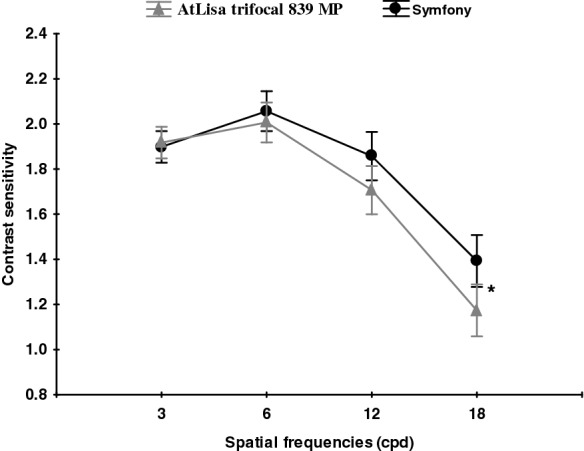
Mean binocular distance contrast sensitivity under mesopic conditions in both groups. A comparison between the Symfony (round black points) and Trifocal (triangular grey points) lenses under mesopic conditions. All data are presented as a mean (points) and 95% confidence interval (whiskers). *Significant difference (*p* < 0.05)

**Fig. 5:**
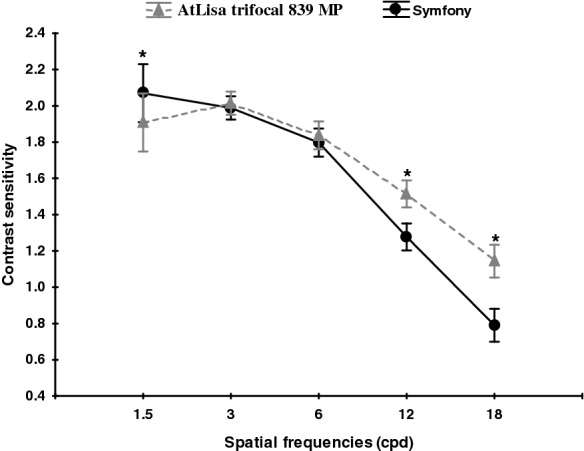
Mean binocular near contrast sensitivity under photopic conditions in both groups. A comparison between the Symfony (round black points) and Trifocal (triangular grey points) lenses under photopic conditions. All data are presented as a mean (points) and 95% confidence interval (whiskers). *Significant difference (*p* < 0.05)

Figures 3, 4 and 5 present mean binocular distance contrast sensitivity under photopic and scotopic conditions and mean binocular near contrast sensitivity under photopic conditions in both groups.

### Photic phenomena

Glare and halo perception was analysed on a 4-point scale (1—none, 4—a lot). Halo perception was more common (4 subjects/20% vs. 1 subject/5%) and more severe (mean score 2.5 ± 0.53 vs. 0.89 ± 0.13; *p *< 0.000) in AtLisa tri 839 MP group than in the Symfony group. Four subjects (20%) in AtLisa tri 839 MP group and only one patient (5%) in the Symfony group reported glare effect. The mean level of glare perception was significantly lower in the Symfony group (0.5 ± 0.69 vs. 2.5 ± 0.53, *p* < 0.000).

### Patient questionnaire

Table 1 shows a comparison of responses to individual items on the modified VFQ-25 in both groups (scale 1–5; 1—the best, 5—the worst).

**Table 1:** Modified VFQ-25 scores in both groups

		AtLisa tri 839 MP group	Symfony group	*p*
1. Reading newspaper	(1–5)	1.35 ± 0.46	1.78 ± 0.71	> 0.05
2. Using computer	(1–5)	1.44 ± 0.50	1.17 ± 0.37	> 0.05
3. Seeing signs	(1–5)	1.05 ± 0.23	1.00 ± 0.00	> 0.05
**4. Daytime driving**	**(1**–**5)**	**1.00 ± 0.00**	**1.21 ± 0.36**	**0.002**
**5. Night driving**	**(1**–**5)**	**1.57 ± 0.55**	**2.13 ± 1.15**	**0.027**
**6. Difficult situation driving**	**(1**–**5)**	**1.14 ± 0.31**	**1.53 ± 0.56**	**0.049**
7. Seeing up close	(1–5)	1.33 ± 0.47	1.78 ± 0.71	> 0.05
8. Steps and stairs	(1–5)	1.00 ± 0.00	1.11 ± 0.46	> 0.05
9. General VA quality	(1–5)	1.85 ± 0.50	1.67 ± 0.47	> 0.05

The overall vision quality and parameters ascertained by items 2, 3, 7 and 8 were satisfying and comparable in both groups

Significant differences between AtLisa tri 839 MP and Symfony groups are indicated by bold letters and numbers

However, the scores for daytime driving (1.00 ± 0.00 vs. 1.21 ± 0.36; *p* = 0.002), night driving (1.57 ± 0.55 vs. 2.13 ± 1.15; *p* = 0.027) and difficult situation driving (1.14 ± 0.31 vs. 1.53 ± 0.56; *p* = 0.049) were significantly better in the AtLisa tri 839 MP group than in the Symfony group.

### Postoperative complications

No postoperative complications were noted over the 1-year follow-up in both groups. There was no case of posterior capsule opacification, which could reduce visual acuity, and there was no need for the Nd:Yag laser capsulotomy.

## Discussion

The comparison of visual outcomes after bilateral implantation of trifocal IOLs (AtLisa tri 839 MP) and extended range of vision intraocular lenses (Symfony) presented in this study demonstrated comparable, promising visual outcomes in both groups. The obtained results were similar to the UDVA findings presented in other studies comparing Symfony and trifocal IOLs, i.e. Fine Vision, Panoptix [8–14]. To date, only one study comparing outcomes after Symfony and AtLisa tri 839 MP [11] has been published. In comparison with it, though, the follow-up of 12 months in our study is significantly longer.

In our prospective case series, binocular UIVA at three distances and UNVA were significantly worse in the Symfony group than in the AtLisa tri 839 MP group. However, this difference was not reflected in patient-reported scores on the modified VFQ-25. This relationship was seen in the study by Ruiz–Mesa et al. [13] but only for binocular UNVA. In our study, the tendency for better binocular UIVA at 66 cm was also detected. In our study, the defocus curve characteristic for AtLisa tri 839 MP and for Symfony IOLs was in line with those described by Mojzis et al. [7] and by the manufacturer (http://www.precisionlens.net/tecnis-symfony-extended-depth-of-focus, 2016). The between-group defocus curve comparison revealed a significant near VA reduction in the Symfony group (ranging from − 2.5 to − 4.0 D). Less obvious differences were observed for intermediate distance and distance VA, where the results were significantly better in the Symfony group, although the magnitude of difference was relatively small. The defocus curve shape in our study was approximately in agreement with that reported by Ruiz-Mesa et al. [9, 13] where other trifocal IOLs (FineVision and Panoptix) offered significantly better near VA than Symfony IOLs. The distance, intermediate distance and near VA measured using standard tests were comparable in both groups.

All subjects in the AtLisa tri 893 MP IOL group were spectacle independent. Whereas the defocus curve analysis revealed significantly worse near vision in the Symfony group, only two patients from this group needed additional near correction. In the VFQ-25, the AtLisa tri 839 MP group reported better near vision, although this difference was not significant. A comparison of visual acuity at different distances between Fine vision and Symfony IOLs [9] as well as Panoptix and Symfony IOLs [8, 9] yielded very similar results.

The distance contrast sensitivity was measured under photopic and mesopic conditions, and near contrast sensitivity was measured under photopic conditions in both groups. Assessed under photopic conditions, the distance contrast sensitivity was in the normal range [16] for all spatial frequencies in both groups, with the tendency to lower values for higher spatial frequencies. This was also observed in other studies comparing CS after bilateral implantation of Symfony and trifocal IOLs [13]. In our study (CSV-1000), the binocular distance contrast sensitivity under photopic conditions was almost the same in both groups. However, when measured under mesopic conditions, the Symfony group achieved significantly better values at the spatial frequency of 18 cpd. In study by Mencucci et al. [11], the Symfony also provided significantly better mesopic CS outcomes than the AtLisa tri 839 MP. According to authors of this study, this higher CS performance may be due to the compensation of the chromatic and spherical aberrations [17, 18]. However, it was not established whether it is due IOL design or other factors, i.e. pupil size in different photic conditions. The study by Eppig et al. evaluated contrast sensitivity in 11 different aspheric IOL designs and concluded that mesopic vision benefits more in aspheric IOL than photopic vision, especially due to the correction of spherical aberration at large pupil diameters, which is supported by the findings of Crnej [19, 20]. Interestingly, both Symfony and AtLisa tri 839 MP have larger diffractive zone (6.0 mm) than PanOptix (4.5 mm) and lower energy utilization (85–86%) than PanOptix (up to 88%) which makes functional vision to be more dependent on pupil size or lightning conditions and provides worse CS [14]. The binocular near CS measured under photopic conditions (FACT) was significantly better in the AtLisa tri 839 MP group at higher spatial frequencies (12, 18 cpd). The rational explanation of our CS results being better than those reported by Ruiz–Mesa et al. [13] is the binocular CS measurement and the associated binocular summation. The study published by Kretz et al. [21] demonstrated that binocular implantation of diffractive trifocal IOLs (AtLisa tri 839 MP) provides significant VA improvement for all distances and fully functional vision including functional stereopsis, as compared to monocular visual outcome. The 12-month follow–up (longer than in other studies) and longer neuroadaptation time may have contributed to superior CS results in our study [22–26]. Besides neuroadaptation, the other possible explanation for better near CS outcome in AtLisa tri 839 MP group might be major vergence dependence of this IOL as described by Esteve-Taboada [26]. In this study, the optical quality (under the large aperture—4.5 mm) of Symfony IOL and AtLisa tri 839 MP IOL was described through modulation transfer function (MTF), the Strehl ratio, cut-off frequency, area of visibility [26]. All metrics revealed that Symfony IOL showed the best optical quality at intermediate vision and the AtLisa tri 839 MP at near vision. The Symfony IOL showed better results than trifocal IOL at − 2.00 and − 2.50 D of vergence, while AtLisa tri 839 MP showed better outcome at − 3.00 and − 3.50 D vergence [26].

The incidence and perception level of halo and glare were significantly lower in the Symfony group. One possible explanation for this finding is that by correcting corneal chromatic and spherical aberrations, the Symfony IOL creates sharper light focus [27] and its echelette design extends the range of vision. Unlike the AtLisa tri 839 MP IOL, the Symfony IOL does not have diffractive steps. However, a high number of diffractive steps/edges are known to be responsible for glare and halo. According to Monaco et al. [8] and Sudhir et al. [14], a relatively higher frequency or greater degree of bother of photic phenomena (glare, halo) is reported with Symfony IOL than with trifocal IOLs and PanOptix IOL. Ruiz-Messa et al. [13] did not find significant differences in halometry between patients after Symfony and FineVision IOL implantation. A possible explanation for this may stem from the fact that Fine Vision has fewer diffractive steps than other diffractive IOLs (for example, Tecnis-32 steps, or AtLisa tri 839 MP—29 steps), and its diffractive steps are convoluted with smoothed edges, which attenuates halo effect in a manner similar to Symfony IOLs. It is difficult to get conclusive incidence of photic phenomena, because many of IOL studies use non-validated questionnaires to capture patient-related outcomes and variation in questionnaires is also used. Unfortunately, this is limitations of our study as well.

The visual outcome assessment findings were approximately consistent with the VFQ-25 scores. Overall satisfaction was very high in both groups. Despite higher incidence of photic phenomena such as halo and glare, subjects after AtLisa tri 839 MP IOL implantation reported significantly better driving comfort than those after Symfony IOL implantation (*p *< 0.05). This might be due the better objective outcomes at distance vision for AtLisa tri 839 MP IOL in comparison with the Symfony IOL [2]: average MTF, higher cut-off frequency (vergency 0.00 D: 163 vs. 146 cycles/mm, respectively), greater area of visibility (vergency 0.00 D; 0.38 vs. 0.22, respectively). In the same study, Strehl ratio (vergency 0.00) for trifocal IOL was 0.34 while for Symfony IOL 0.02. In the VFQ-25, the patients with trifocal IOLs significantly better assessed both their daytime and night-time driving comfort. Moreover, better driving scores in VFQ-25 could be explained by the fact that after 1 year of neuroadaptation, glare and halo effects were no longer perceived by the patients as detrimental for driving. However, this hypothesis would require further research in a larger sample with a longer follow-up.

The Symfony IOL was implanted through a mean 2.2-mm clear corneal incision and the AtLisa tri 839 MP IOL through a mean 1.8-mm clear corneal incision. In our opinion, the size of corneal incision did not significantly affect the assessed visual parameters. It is commonly known that the size of corneal incision affects the surgically induced astigmatism (SIA). However, it is only true for 2.2–3-mm corneal incisions [28–30]. The difference in SIA is negligible in eyes with corneal incision below 2.2 mm [30, 31]. Furthermore, in corneal incisions smaller than 3.5 mm, the SIA differences are only significant over the short-term postoperative period and they decrease with the longer follow-up. In the study by Kim et al., there was no significant difference in SIA between the 1.8-mm and 2.2-mm clear corneal incision (0.21 D vs 0.29 D) [32]. In the study by Yang et al. [33] with a 6-week follow-up, the SIA with the clear corneal incision of 1.8 mm and 2.2 mm was 0.25 ± 0.1 D and 0.27 ± 0.1 D, respectively. The differences, however, were not significant. Furthermore, the review by Dewey et al. [34] demonstrated lack of unambiguous evidence to support significant differences in SIA in eyes with corneal incision < 2.6 mm. Therefore, it seems that combining two subgroups (clear corneal incision 1.8 mm and 2.2 mm) did not significantly affect postoperative visual function, rates of complications and photopic phenomena, i.e. glare or halo.

To date, only one study directly comparing visual outcomes after AtLisa tri 839 MP and Symfony implantation has been published [11]. However, it reported a 3-month follow-up only, compared to 1 year in our study. The presented results indicate that Symfony IOL implantation is associated with lower incidence of postoperative visual disturbance and its lower subjective perception level. It should be noted that the differences in photic phenomena (halo and glare) between Symfony and AtLisa IOLs do not correspond to the patient-reported satisfaction with their visual function, including driving comfort. The results of our study strongly suggest that both IOLs AtLisa tri 839 MP and Symfony are good options for many patients who require spectacle-free vision for their lifestyle. While both IOLs provide satisfying distance and intermediate distance visual acuity, AtLisa tri 839 MP IOL offers better near vision. The multi-centre study by the Concerto group [35] demonstrated that the implantation of Symfony IOLs with micromonovision (dominant eye–target refraction–emmetropia, fellow eye–low myopia of − 0.75 D) provides significantly better uncorrected intermediate and near visual acuity than non-monovision technique. However, patient-reported visual function assessed using the VFQ-25 in our study (and, in particular, no difference in near vision quality between Symfony and AtLisa tri 839 MP) seems to support emmetropia as binocular target refraction in eyes implanted with Symfony lenses. In our opinion, binocular emmetropia might be a reasonable alternative to micromonovision in this group of patients.
